# Incidentally detected double-chambered right ventricle in an adult

**DOI:** 10.1016/j.radcr.2026.06.159

**Published:** 2026-07-17

**Authors:** Thomas Saliba, David Rotzinger, Guillaume Fahrni, Philippe Thoma

**Affiliations:** a Hopital Erasme, Université Libre de Bruxelles, Brussels, Belgium; b Centre Hospitalier Universitaire Vaudois (CHUV), University of Lausanne, Lausanne, Switzerland

**Keywords:** Double-chambered right ventricle, Congenital heart disease, Right ventricular obstruction, Cardiac MRI, Echocardiography, Adult congenital heart disease

## Abstract

Double-chambered right ventricle (DCRV) is a rare congenital cardiac anomaly caused by hypertrophy of right ventricular muscular structures, most commonly the moderator band, resulting in intracavitary obstruction and division of the right ventricle into high- and low-pressure chambers. It accounts for approximately 0.5%-2% of congenital heart diseases and is usually diagnosed in childhood, though delayed presentation in adulthood may occur. DCRV is frequently associated with other congenital anomalies and is often discovered on cardiac imaging. A 55-year-old asymptomatic woman was referred for cardiology evaluation due to a family history of thoracic aortic aneurysms. Physical examination revealed pectus excavatum and a cardiac murmur. Electrocardiography showed left ventricular hypertrophy with repolarization abnormalities. Transthoracic echocardiography identified right ventricular apical obstruction, prompting cardiac MRI. This demonstrated marked right ventricular hypertrophy and a prominent moderator band dividing the ventricle into proximal and distal chambers, consistent with type 1 DCRV. Cine imaging revealed a focal high-velocity jet at the obstruction site. The patient was referred for cardiology follow-up to consider surgical management, though this was not found to be necessary due to her being asymptomatic. DCRV is diagnostically challenging due to nonspecific or absent symptoms in adults. While echocardiography may detect obstructive flow, cardiac MRI is essential for detailed anatomical assessment and identification of associated anomalies. Early diagnosis is important, as guidelines recommend surgical intervention to prevent right ventricular dysfunction. DCRV may present in asymptomatic adults. This case highlights the value of advanced cardiac imaging in diagnosing atypical right ventricular obstruction and guiding timely management to prevent disease progression.

## Introduction

Double-chambered right ventricle (DCRV) is a rare congenital cardiac anomaly which presents with a hypertrophied moderator band [1]. This hypertrophied moderator band causes an intracavitary obstruction of the right ventricle, dividing it into a high pressure chamber near the tricuspid valve and a lower pressure chamber near the pulmonary valve [2]. This anomaly remains uncommon, only accounting for 0.5%-2% of all congenital heart diseases [3]. Patients are generally diagnosed in childhood or adolescence, though some reach adulthood without the diagnosis being made [3]. It is important to make the diagnosis of DCRV as such patients can also often have associated anomalies such as ventricular septal defects, pulmonary valve stenosis, Tetralogy of Fallot, double outlet right ventricle, Ebstein's anomaly or subaortic stenosis [1].

The diagnosis is often incidentally made whilst performing imaging for workups of heart murmurs or other cardiac pathologies [4].

We present the case of a 55-year-old woman who presented for a cardiac MRI after being referred by her cardiologist.

## Case report

A 55-year-old woman was referred to cardiology for evaluation due to a family history of thoracic aortic aneurysms. She was asymptomatic. Physical examination revealed pectus excavatum and a grade 4/6 systolic murmur that was difficult to localize. Her blood pressure was 140/80.

A blood test was conducted, with no abnormal findings being reported.

The patient was not known to suffer from any connective tissue syndromes or congenital diseases or syndromes.

The patient had no known relevant medical or surgical history. The patient had no relevant family history other than the thoracic aortic aneurysms.

Electrocardiography demonstrated left ventricular hypertrophy with repolarization abnormalities and a heart rate of 79 beats per minute. Transthoracic echocardiography revealed an apical obstruction of the right ventricle, prompting further evaluation with cardiac MRI.

Cardiac MRI was performed on a 1.5T machine which demonstrated marked hypertrophy of right ventricular (RV) muscular structures, with wall thickness approaching that of the left ventricle (Fig. 1, Fig. 2). A prominent hypertrophied moderator band divided the right ventricle into a large proximal chamber and a smaller distal chamber, consistent with type 1 DCRV (Fig. 3). Retrospective cine True FISP images, acquired in the short axis, long axis, 4-chambre, 3-chambre and right ventricle views, revealed a focal flow void at the site of obstruction, corresponding to a high-velocity jet (Fig. 4, Fig. 5). Phase-contrast images were acquired the plane of the right ventricular outflow tract in order to estimate the degree of stenosis. The right ventricular outflow tract flow showed a peak velocity of 263 cm/s, a peak flow of 160 mL/s and a pressure gradient of 28 mmHg. The pulmonary artery was at the superior limit of the normal range, measured at 29 × 27 mm, likely secondary to chronic high-pressure flow.

**Fig. 1:**
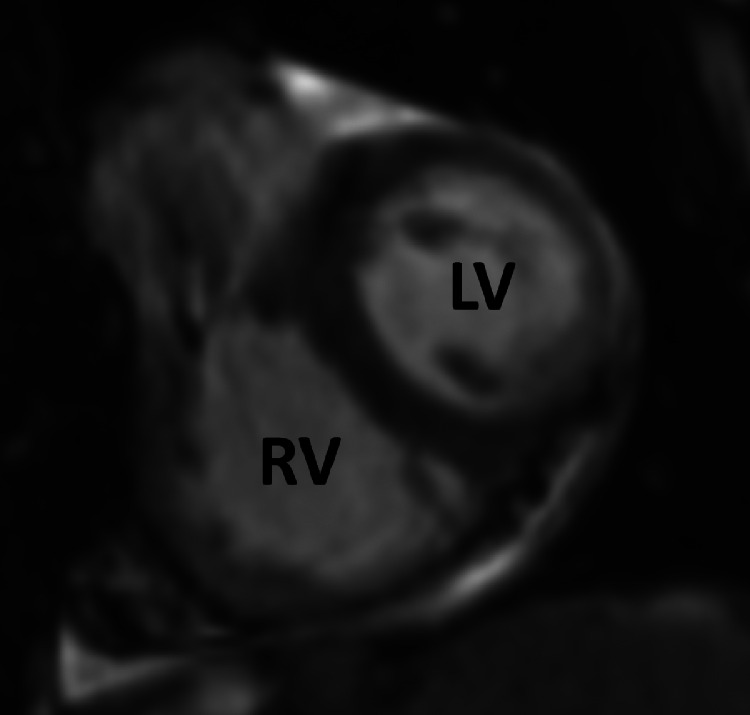
Short axis T2-weighted image showing a right ventricle (RV) with a wall of roughly the same thickness as that of the left ventricle (LV), suggestive of increased intra-ventricular pressure generation in order to eject blood.

**Fig. 2:**
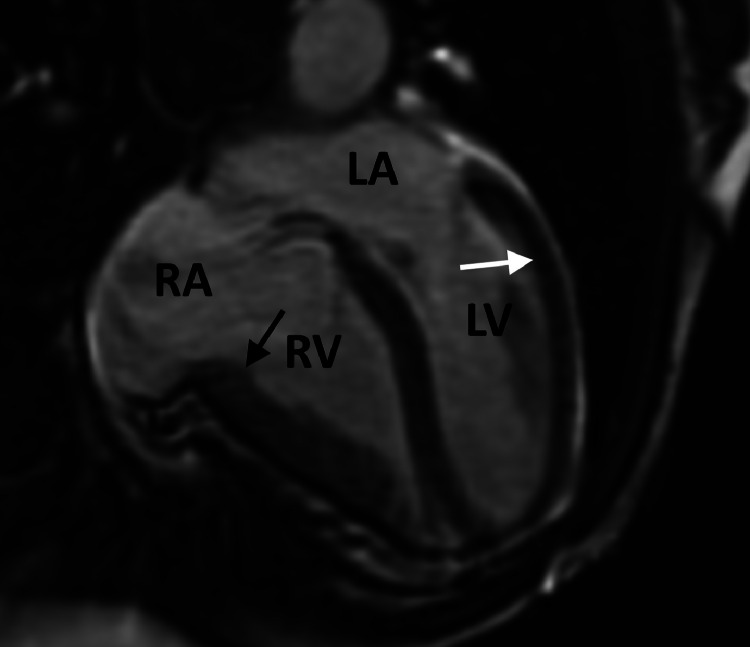
Four-chamber cine view showing the left atrium (LA), right atrium (RA), left ventricle (LV) and right ventricle (RV). The lateral wall of the left ventricle (white arrow) is roughly of equivalent thickness to that of the right ventricle (black arrow).

**Fig. 3:**
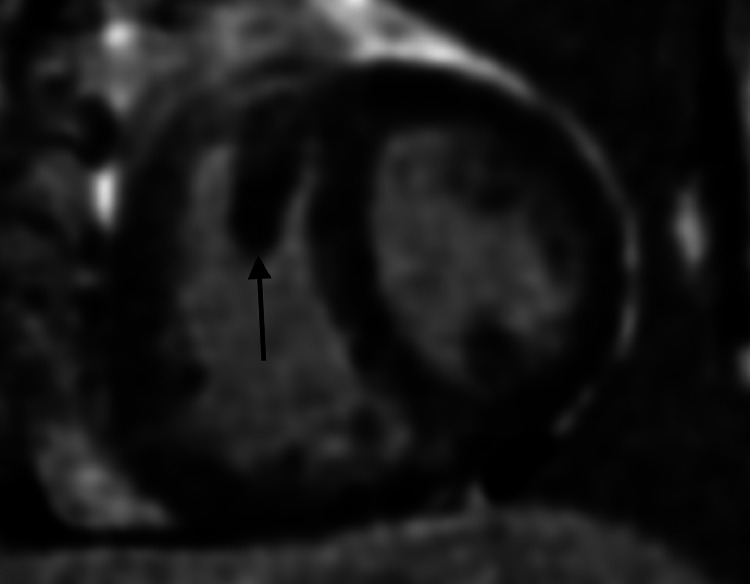
Short axis late gadolinium enhancement image showing a hypertrophied moderator band within the right ventricle, responsible for the obstruction (arrow). The lack of hyperintensity indicates that there is no late gadolinium enhancement of the moderator band.

**Fig. 4:**
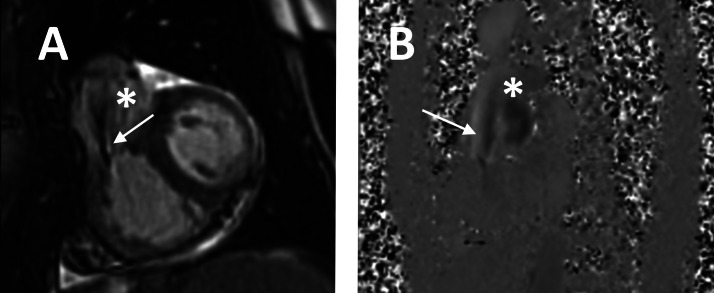
Cine MRI image centered on the right ventricular outflow tract (A) showing the point of stenosis (arrow) with a flow void, signifying locally accelerated blood flow within the proximal pulmonary artery (star). Phase-contrast image (B) in the same plane, clearly showing the rapid blood flow due to stenosis which presents as a hypointensesignal (arrow) within the proximal pulmonary artery (star).

**Fig. 5:**
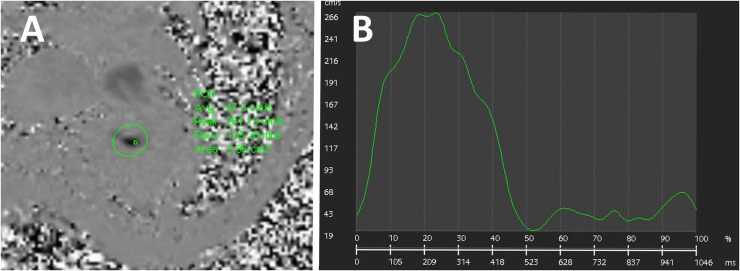
Axial phase-contrast image centered on the aorta (A) showing an acceleration of the flow (green circle) alongside the velocity curve graph (B) showing an acceleration of the flow.

The late gadolinium enhancement acquisition did not show any hyperintense zones within the hypertrophied muscle bundles or the rest of the myocardium.

The left ventricular ejection fraction was 59% (normal: 56%-78%), the end diastolic volume was 100 ml (normal: 58-141 mL), the end systolic volume was 41 mL (normal: 13-51 mL), giving a stroke volume of 59 mL and an estimated cardiac output of 3.51 mL/min.

The right ventricular ejection fraction was 45% (normal: 47%-80%), the end diastolic volume was 113 mL (normal: 58-154 mL), the end systolic volume was 62 mL (normal: 12-68 mL), giving a stroke volume of 50 mL and an estimated cardiac output of 3.03 mL/min.

The patient was referred back to her cardiologist in order to potentially undergo surgical treatment. After undergoing review with the hospital’s multidisciplinary heart team, surgery was not considered necessary at this stage given the patient’s lack of symptoms, with a watchful waiting approach being adopted.

## Discussion

DCRV is a challenging clinical diagnosis due to often atypical symptoms similar to those of other cardiovascular diseases, and is thus often found unexpectedly on imaging [2].

Young patients may be asymptomatic as the hypertrophy of the muscle bands develops over time [3]. Therefore patients typically present as young adults around 30-40 years old with symptoms of dyspnea, thoracic discomfort, dizziness or sports-related syncope [3,5].

DCRV can be subdivided into 2 types. Type 1 DCRV is when the hypertrophied moderator band crosses the right ventricle and is responsible for the obstruction [6]. In type 2 DCRV, there is no hypertrophied moderator band, with the obstruction being caused by parietal and septal muscle hypertrophy [6]. Intraventricular pressure gradients are typically higher in type 1 DCRV compared to type 2, whereas ventricular septal defects (VSD) are more likely to be found in type 2 DCRV [6].

An electrocardiogram can provide a first diagnostic clue by indicating the presence of RV hypertrophy, providing a reason to conduct follow-up imaging [6]. Pectus excavatum is known to cause baseline ECG alterations due to altered positioning of the heart in the chest cavity [7]. In the case of our patient, it is possible that concomitant pectus excavatum was responsible for ECG anomalies being discordant with the patient’s underlying cardiac pathology, though this remains hypothetical.

Echocardiography with doppler is able to reliably detect the presence of high velocity flow around the obstruction, though it is sometimes difficult to see the RV outflow tract [4,5]. On the other hand, cardiac MRI and cardiac CT are important in order to assess the levels of obstruction, ventricular volume, outflow tract and artery dimensions as well as for potential coexisting anomalies [5]. These anomalies often include VSD, which is present in up to 90% of cases according to 1 study [2]. Around 80%-90% of patients will have at least 1 other anomaly, which can include pulmonary valve stenosis, atrial septal defects, aortic or tricuspid regurgitation, Ebstein’s anomaly, tetralogy of Fallot or transposition of the great arteries [2,6,8]. It is therefore essential to search for associated anomalies once the diagnosis of DCRV has been made.

In the case of Ebstein’s anomaly, it can result in a division of the right ventricle if it is large and is angled inferiorly across the inlet and presents with an imperforate membrane or muscular shelf [9,10].

RV outflow tract obstruction is a feature of tetralogy of Fallot, with RV hypertrophy resulting as a consequence in an attempt to overcome the increased pressure [11]. When patients have concomitant unrestrictive ventricular septal defects, the right ventricle overpressure can increase the right-to-left shunting, reducing pulmonary blood flow and worsening the hypoxia [11].

Patients who have undergone corrective surgery for transposition of the great arteries are at risk of developing RV outflow tract obstruction due to the nonphysiological position of the right ventricle [12]. This hypertrophy occurs as an adaptation due to being systemic pump, putting more strain on the right ventricle, with the possibility of subsequent RV outflow tract obstruction [13].

Pathologies such as arrhythmogenic RV cardiomyopathy can also result in RV outflow tract obstruction linked to the thickening of the right ventricle as it undergoes fibrofatty replacement [14]. This can be particularly evident in the early stages, in what was described as the “triangle of dysplasia,” which included the inflow tract, outflow tract, and apex [15].

RV hypertrophy, found in around 30% of patients undergoing cardiac MRI for hypertrophic cardiomyopathy, can also be a cause for RV outflow tract obstruction [16]. In these cases, patients can have variable hypertrophy patterns which can include the septum, apex and free wall are able to cause outflow tract obstruction [16].

In patients presenting with these pathologies, a careful history is needed to do hand-in-hand with the MRI findings in order to establish a diagnosis as findings of right ventricle hypertrophy may be similar.

Current European Society of Cardiology (ESR) guidelines recommend surgery in cases where stenosis is severe, regardless of symptoms [5]. If only surgical valve replacement is possible, it is indicated in patients with severe stenosis when they also present with symptoms [5]. The ESR also recommends surgical valve replacement in patients with severe stenosis who are asymptomatic if they have decreased exercise capacity, decreasing right ventricle function or tricuspid regurgitation, increased right ventricle systolic pressure >80 mmg or right-to-left shunting via an atrial or ventricular septal defect [5]. The ESR even in cases where the pressure gradient is low if they have symptoms related to pulmonary stenosis, decreased RV function or moderate tricuspid regurgitation or right-to-left shunting, though this is a class IIa recommendation [5]. Additionally, patients with pulmonary stenosis should avoid high intensity sports, though pregnancy is generally well tolerated unless the RV outflow tract obstruction or the RV failure is severe [5]. As these patients will require lifelong follow-ups, it is important that their individual cases be considered on a case-by-case basis [5]. The American Heart Association also recommends surgery in patients who are symptomatic or have outflow tract obstruction in order to improve symptoms and prevent adverse remodeling of the right ventricle [17]. Although surgery is often appropriate, patients face the risk of death from low cardiac output or heart failure, even in cases of successful operations [18]. It is also possible to miss ventricular septal defects, which can be shaded by the hypertrophied muscle, in which case the patient may need to be re-operated if the defect is significant [10].

Early surgical correction is recommended as untreated patients tend to deteriorate [19]. If surgery is refused, patients require follow-up due to the risk of low ventricular output, ischemic heart disease, arrhythmia and cardiogenic sudden death [19]. However, some studies have shown that, even without surgery, some patients can remain asymptomatic and clinically stable [8,20]. In the case of our patient, the choice to operate…. (did they operate?).

## Conclusion

DCRV is a rare congenital cardiac anomaly that is frequently associated with other structural defects. Although most patients are diagnosed early in life, delayed presentation into adulthood can occur, particularly in asymptomatic individuals. DCRV is often discovered incidentally during imaging performed for unrelated or nonspecific cardiac findings. Once identified, surgical management is generally recommended to prevent symptom progression and RV dysfunction. This case highlights the importance of advanced cardiac imaging in the diagnosis of atypical RV obstruction in adults.

## Patient consent

We hereby declare that we the patient provided informed consent that their data could be used for scientific purposes.
